# MRI findings in chronic exertional compartment syndrome of the forearm: Using signal intensity ratio as a diagnostic tool

**DOI:** 10.4102/sajr.v25i1.2219

**Published:** 2021-10-06

**Authors:** Jacques Badenhorst, Mark Velleman, Audrey Jansen van Rensburg, Tanita Botha, Nikki van der Walt, Christa Janse van Rensburg

**Affiliations:** 1 Department of Diagnostic Radiology, Faculty of Health Sciences, University of Pretoria, Pretoria, South Africa; 2 Radiologist, Private Practice, Capital Radiology, Pretoria, South Africa; 3 Section Sports Medicine, Faculty of Health Sciences, University of Pretoria, Pretoria, South Africa; 4 Department of Statistics, Faculty of Natural and Agricultural Sciences, University of Pretoria, Pretoria, South Africa; 5 Orthopaedic Specialist, Private Practice, Unitas Hospital, Pretoria, South Africa

**Keywords:** forearm compartment syndrome, chronic exertional compartment syndrome, CECS, exercise MRI, forearm pain, compartment syndrome, athletes

## Abstract

**Background:**

Chronic exertional compartment syndrome (CECS) of the forearm is a rare but important cause of morbidity amongst athletes involved in strenuous upper limb activities. The diagnosis remains challenging due to the absence of objective, reproducible diagnostic studies.

**Objectives:**

To assess and quantify signal intensity (SI) changes of involved muscles in patients with CECS of the forearm compared to healthy control subjects competing in similar sporting disciplines. Also, to objectively measure MRI SIs within muscle compartments when using a pre- and post-exercise regime and calculating a signal intensity ratio (SIR) between post- and pre-exercise studies.

**Method:**

The study retrospectively examined MRI scans of patients treated for CECS of the forearm and compared these to the MRI scans of asymptomatic high-level rowers. A specific, reproducible pre- and post-exercise MRI scanning protocol was utilised in both patient and control subjects between 2011 and 2020. Signal intensities were evaluated pre- and post-exercise in involved muscle groups and ratios were calculated.

**Results:**

A total of 86 SIs were measured (43 pre- and 43 post-exercise) in nine study participants (five patients and four controls). After post:pre-exercise comparisons, a statistically significant difference was found between control and patient groups (*p* = 0.0010). The extensor carpi radialis, flexor digitorum profundus and flexor digitorum superficialis muscles were most commonly involved.

**Conclusion:**

This study confirms that significant SI changes are apparent in patients with CECS of the forearm when making use of a standardised pre- and post-exercise MRI protocol. Furthermore, SIR may be used to accurately diagnose CECS of the forearm.

## Introduction

Chronic exertional compartment syndrome (CECS) of the forearm is a rare entity with the first case only being described in 1983.^1^ Most of the literature regarding this condition is in the form of case studies or case series, and mostly include patients such as rowers, motorcyclists, rock-climbers, resistance trainers, etc., who undertake strenuous, repetitive forearm movements.^2,3,4,5,6,7,8,9,10,11,12,13,14^ Symptoms in patients with CECS typically occur during exertion and resolve with rest. Symptoms include pain, paraesthesia, cramping, a ‘stiff’ feeling of the involved compartment as well as loss of grip strength.^2,4,7,8,10,11,12,14,15^ The pathophysiology of the condition remains incompletely understood but is based on the premise of raised intra-compartmental pressures within a relatively rigid fascial compartment, causing reduced tissue perfusion and subsequent ischaemia.^2,8,15,16,17,18,19,20^

During exercise, it is normal for intra-compartmental pressures to rise; however, this increased pressure does not usually cause symptoms or altered tissue oxygenation as in patients with CECS.^2,9,19^ Patients with CECS have relatively greater increases in pressure and these pressures also take longer to return to normal after cessation of exercise.^21^ Furthermore, there is evidence that patients with CECS have relatively low levels of oxygenation within muscle compartments during exercise and recovery, as well as deoxygenation early on in exercise, before intra-compartmental pressures rise significantly.^18^ This points to altered oxygen extraction from the blood by affected muscles as well as altered muscle metabolism, likely because of a chronic state of low-grade ischaemia during exercise.^18^

Despite the growing amount of available literature on CECS, controversy remains on how best to diagnose the condition, as well as what diagnostic parameters to use within each specific modality.^1,2,3,7,10,12,22,23^ Currently, the most widely used method is based on intra-compartmental pressure testing and the values proposed in 1990.^21^ These values were proposed for lower leg CECS, and various modifications of the values have been suggested for diagnosis of forearm CECS; however, none are universally accepted.^8,12,24^ Various studies confirmed that a significant proportion of patients with clinical and surgically responsive CECS may be misdiagnosed with the use of current intra-compartmental pressure values.^10,12,23,24^ Moreover, there is an ever-changing variation in the proposed ‘normal’ compartmental pressure values of the forearm.^1,23,25^ Furthermore, the invasive nature of testing, the associated risks and the lack of an accepted testing technique make intra-compartmental pressure manometry a far from ideal diagnostic tool.^7,10,12,17,18,20,23,26^

MRI in chronic compartment syndrome is based on the premise that ischaemic muscle will become oedematous and thus exhibit a more intense T2-weighted (T2W) signal compared with normal muscle.^15,17,26^ Additionally, MRI can accurately exclude other conditions that may mimic CECS, for example, tendonitis, muscle tears and nerve impingements. MRI scanning has been utilised for the past 30 years in the diagnosis of CECS, with numerous studies and case reports published at testing to its usefulness.^4,9,13,16,17,19,26,27,28,29^

Despite the relatively strong evidence for the use of a pre-post exercise MRI protocol in CECS, no standardised methods regarding exact exercise protocols and imaging acquisition have been published.^6,15,17,26,28,30^ Furthermore, in the absence of T2W mapping software, signal intensity (SI) is graded subjectively, presenting the possibility for greater inter-observer variations.^6^ The ever-increasing competitiveness and demands faced by top-level and professional athletes require that timeous and accurate diagnosis of any sporting-related symptoms is paramount to the longevity and success of an athlete. In our setting, several rowers have been treated for CECS, necessitating the need for future accurate, reproducible and reliable diagnostic methods.

This study aimed to quantify SI changes in patients with CECS of the forearm and compare them with healthy control subjects participating in similar sporting disciplines. A further aim was to objectively measure MRI SI within muscle compartments when using a pre- and post-exercise regime and calculate a signal intensity ratio (SIR) between post- and pre-exercise studies.

## Methods

### Study design

The study makes use of a cross-sectional descriptive pilot study design. Study findings in a CECS patient group were compared to a control group.

### Setting

A private radiology practice based at both the Life Groenkloof Hospital (part of Pretoria MR Trust) and the Sport, Exercise Medicine and Lifestyle Institute (SEMLI), University of Pretoria, South Africa was the setting for this study. Sport, Exercise Medicine and Lifestyle Institute is located at the High-performance Centre^31^ in Pretoria, South Africa, which is a high-level specialist sporting centre and served as training base for all our control subjects. Patients were assessed by an experienced upper limb orthopaedic surgeon in private practice.

### Study population and sampling strategy

Patients (*n* = 5) included in the study all received fasciotomy surgery for treatment of CECS of the forearm, with symptoms improving or resolving after surgery. Patients who underwent concurrent nerve release surgery, had a history of additional previous surgery to the forearm, or who had a history of significant trauma to the forearm, were excluded Patients all underwent MR imaging at the above centres between 2009 and 2020.

The control subjects (*n* = 4) were athletes enrolled in the University of Pretoria Rowing Team and included individuals who were symptom-free when rowing, did not undergo any previous forearm surgery and had not previously sustained significant trauma to the forearms. Control subjects were screened for eligibility by completing a questionnaire that excluded any of the above conditions.^32^

### Evaluation procedure

A novel, custom pre- and post-exercise scan protocol was followed to reproduce CECS symptoms in both patient and control subjects. Initially, an MRI scan was performed on all participants (patients and controls) at rest. They were then asked to do a repetitive exercise (concentric hand squeezes of a foam ball up to the point of symptom onset or exhaustion), after which the forearm(s) was immediately scanned again.

Patient scans were performed on either a 1.5-T MRI scanner (1.5T) or a 3-T MRI scanner (3T). Control subjects were scanned on a 3T scanner. Both 1.5-T and 3-T scanners were used because of the equipment changes during the research period, as well as different machines being used at the different imaging sites.

The patient group was imaged using fat-sensitive T1-weighted (T1W) sequences as well as fluid-sensitive T2W sequences with fat-suppression short-tau inversion recovery (STIR).

Control subjects only underwent fat-suppressed T2W (STIR) imaging both before and after exercise. As a result of the time constraints in the MRI suite, the control group had their forearms scanned simultaneously. This resulted in differing imaging parameters and a modest decrease in image resolution. The sequences used are listed in Table 1.

**TABLE 1:** MRI sequences obtained in patients and control groups.

Study Group and MRI magnet strength	Pre-exercise		Post-exercise	
	Sequence	Metrics	Sequence	Metrics
Patient 1.5T	T1W	Axial (TE 25, TR 664, Slice thickness 4 mm, gap 10%, 70 slices) Coronal (TE 25, TR 4500, Slice thickness 3 mm, gap 10%, 21 slices)	STIR (T2W)	Axial (Same as pre-exercise)
	STIR (T2W)	Axial (TE 29, TR 7680, Slice thickness 4 mm, gap 10%, 70 slices Sagittal (TE 29, TR 4500, Slice thickness 3 mm, gap 10%, 29 slices) Coronal (TE 29, TR 4500, Slice thickness 3 mm, gap 10%, 21 slices)		
Patient 3T	T1W	Axial (TE 20, TR 500-800, Slice thickness 3 mm, gap 1 mm, 50 slices) Coronal (TE 15, TR 692, Slice thickness 3 mm, gap 1 mm, 17 slices)	STIR (T2W)	Axial (Same as pre-exercise)
	STIR (T2W)	Axial (TE 60, TR 8583, Slice thickness 3 mm, gap 1 mm, 50 slices) Coronal (TE 30, TR 3194, slice thickness 3 mm, gap 0.5 mm 13 slices) Sagittal (TE 30, TR 5021, Slice thickness 3 mm, gap 0.5 mm, 20 slices)		
Control 3T	STIR (T2W)	Axial (TE 60, TR 10300, Slice thickness 3 mm, 60 slices)	STIR (T2W)	Axial (Same as Pre-exercise)

TE, time to echo; TR, time to repetition; 1.5T, 1.5-Tesla MRI scanner; 3T, 3-Tesla MRI scanner; STIR, Short Tau Inversion Recovery; T2W, T2 weighted.

## Data capturing

Anonymous MRIs were evaluated using dedicated radiological workstations and AGFA picture and archiving system (PACS) for both patient and control subjects. Muscles that exhibited an increase in STIR signal on post-exercise contrast imaging were further investigated by placing a circular region of interest (ROI) within the respective muscles for both the post-exercise and the corresponding pre-exercise STIR images. Region of interests used were either 10 mm or 5 mm in diameter depending on the size of the area suitable for measurement. Tendons, vascular structures, nerves and surrounding fat were excluded from the ROI. Absolute SI was measured both pre- and post-exercise, and results were entered into an Microsoft Excel data table alongside the corresponding arm, age and sex of the participant.

Muscles involved were also recorded and included extensor carpi radialis (ECR), extensor carpi ulnaris (ECU), extensor digitorum (ED), brachioradialis (BR), supinator (Sup), abductor pollicis (AP), flexor digitorum profundus (FDP), flexor digitorum superficialis (FDS), flexor pollicis longus (FPL) and flexor carpi radialis (FCR). Findings were validated by a musculoskeletal radiologist with more than 20 years of experience.

Moreover, the pre- and post-exercise SI of involved muscles was compared, and a post:pre-exercise SIR was calculated. This ratio was obtained by dividing the measured SI of the post-exercise study by the measured SI of the pre-exercise study.

### Statistical analysis

Demographic data including age and sex of participants were analysed as were the arms and muscles affected. Descriptive statistics included measurements such as mean, standard deviations, frequencies and proportions to describe the results. Tabulated and graphical representations were made where applicable to assist in visualising aspects of the data. The Shapiro–Wilk test was used to determine whether the data were normally distributed, followed by the independent t-test that investigated whether a significant difference existed between the SIR values in the control and patient groups. All significance tests were performed at a 5% level of significance.

## Results

**FIGURE 1:**
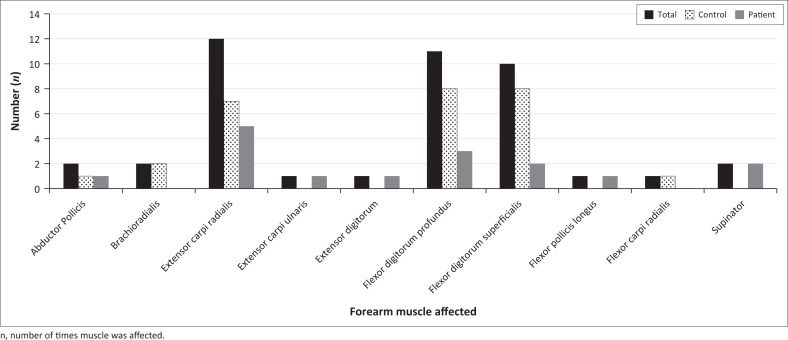
Individual muscles involved per record.

The participant group consisted of a total of four (44.4%) males and five (55.6%) females. Demographics of all study participants, as well as the arm(s) that exhibited SI changes on post-exercise imaging, are detailed in Table 2 and Figure 1. As both arms were involved in some individuals, the sum of individual arms affected is more than the total number of participants. The number of muscles involved per sex and arm are listed in Table 3.

**TABLE 2:** Demographic profile of participants by age, sex and arm(s) involved.

Demographic parameter	All participants (*n* = 9)			Control group (*n* = 4)			Patient group (*n* = 5)		
	*n*	%	Mean ± s.d.	*n*	%	Mean ± s.d.	*n*	%	Mean ± s.d.
**Age (years)**	-	-	20.11 ± 4.37	-	-	20.25 ± 1.89	-	-	20.00 ± 5.96
**Sex**									
Male	4	44.4	-	0	0.0	-	4	80	-
Female	5	55.6	-	4	100.0	-	1	20	-
**Arm**									
Right	7	77.8	-	4	100.0	-	3	60.0	-
Left	6	22.2	-	4	100.0	-	2	40.0	-

*N*, number; %, percentage of total participants; s.d., standard deviation.

**TABLE 3:** Number of muscles involved by sex and arm.

Demographic parameter	Total muscles (*n* = 43)		Control (*n* = 27)		Patient (*n* = 16)	
	*n*	%	*n*	%	*n*	%
**Sex**						
Male	13	30.2	0	0.0	13	81.2
Female	30	69.8	27	100.0	3	18.8
**Arm**						
Right	22	51.2	13	48.1	9	56.2
Left	21	48.8	14	51.9	7	43.8

*N*, number; %, percentage of total records.

A total of 86 SIs were collected (43 pre-exercise and 43 post-exercise measurements). The SI in 43 different muscles across the control (*n* = 27; 62.8%) and patient (*n* = 16; 37.2%) groups was measured. Muscle SI values were collected in the right arm (*n* = 22; 51.2%) and the left arm (*n* = 21; 48.8%) of males (*n* = 13; 30.2%) and females (*n* = 30; 69.8%).

The number of specific muscles involved are listed in Table 4 and Figure 1. Muscles involved were predominantly the ECR (*n* = 12; 27.9%), FDP (*n* = 11; 25.6%) and FDS (*n* = 10; 23.3%). In the patient group, the ECR (*n* = 5; 31.2%) followed by the FDP(*n* = 3; 18.8%) was mostly involved. In the control group, FDP and FDS were equally involved (*n* = 8; 29.6% each) followed by ECR (*n* = 7; 25.9%).

**TABLE 4:** Specific muscles involved.

Muscles affected	All (*n* = 43)		Control (*n* = 27)		Patient (*n* = 16)	
	*n*	%	*n*	%	*n*	%
Abductor pollicis (AP)	2	4.7	1	3.7	1	6.2
Brachioradialis (BR)	2	4.7	2	7.4	0	0.0
Extensor carpi radialis (ECR)	12	27.9	7	25.9	5	31.2
Extensor carpi ulnaris (ECU)	1	2.3	0	0.0	1	6.2
Extensor digitorum (ED)	1	2.3	0	0.0	1	6.2
Flexor digitorum profundus (FDP)	11	25.6	8	29.6	3	18.8
Flexor digitorum superficialis (FDS)	10	23.3	8	29.6	2	12.5
Flexor pollicis longus (FPL)	1	2.3	0	0.0	1	6.2
Flexor carpi radialis (FCR)	1	2.3	1	3.7	0	0.0
Supinator (S)	2	4.7	0	0.0	2	12.5

**FIGURE 2:**
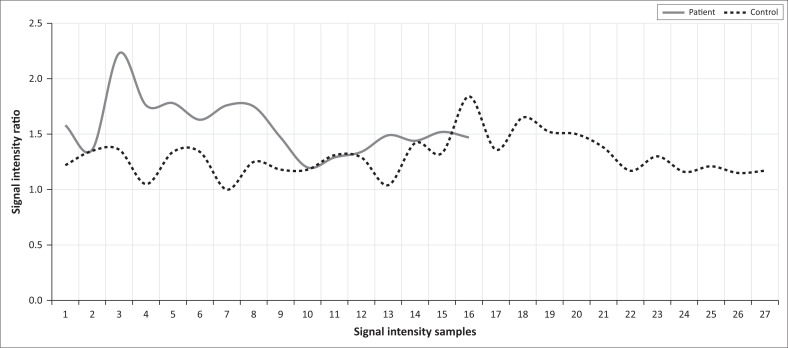
Signal intensity ratio plotted per data record.

**FIGURE 3:**
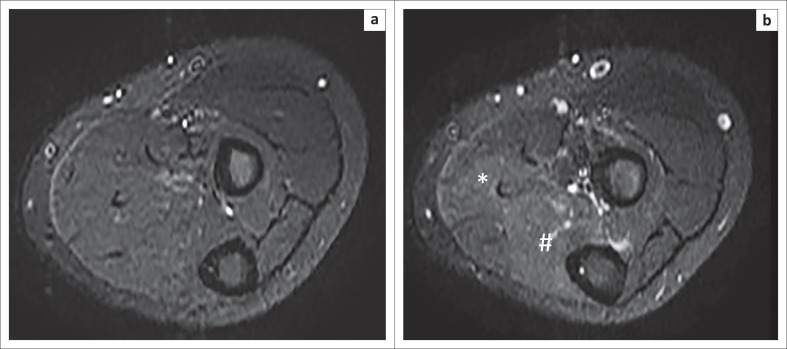
Pre-exercise (a) and post-exercise (b) sequences in a control subject revealing subtle increases in signal within the flexor digitorum superficialis (*) and flexor digitorum profundus (#) muscles.

**FIGURE 4:**
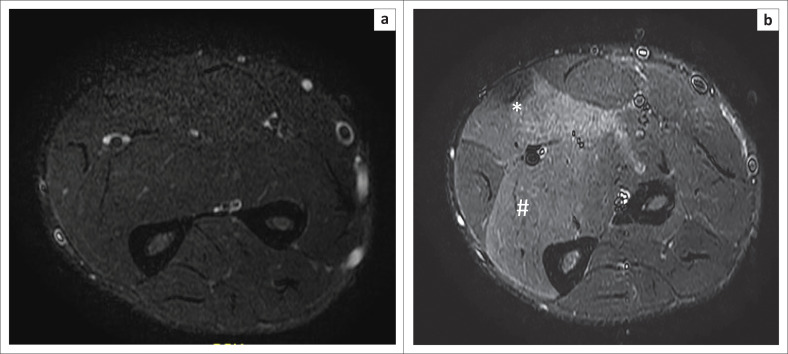
Pre-exercise (a) and post-exercise (b) sequences in a CECS patient demonstrating clear signal increase in the flexor digitorum superficialis (*) and flexor digitorum profundus (#) muscles after exertion.

The post:pre-exercise SIR measurements are depicted in Table 5 and Figure 2. The SIR in the control group had a range between 1.0 and 1.84 whilst for the patient group the SIR range varied between 1.20 and 2.23. There was a statistically significant difference (*p* = 0.0010) in the SIR between the control (1.30 ± 0.18) and patient (1.57 ± 0.25) groups. Examples of pre- and post-contrast imaging studies in both control (Figure 3) and patient (Figure 4) groups are depicted below.

**TABLE 5:** Post:pre-exercise signal intensity ratio measurements.

Post:pre-exercise SIR	All participants (*n* = 43)	Control (*n* = 27)	Patient (*n* =16)	*p*
Min	1.0	1.0	1.2	0.0010*
Max	2.23	1.84	2.23	
Mean ± s.d.	1.40 ± 0.25	1.30 ± 0.18	1.57 ± 0.25	

* , *p* < 0.05.

*N*, number of SIR measurements; %, percentage of total participants; s.d., standard deviation; Min, minimum; Max, maximum.

## Discussion

This study confirms that there are objectively measurable SI changes in the forearm muscles of both control and patient subjects when making use of a pre- and post-exercise imaging protocol. Furthermore, the study illustrated a statistically significant difference in SIR when utilising a pre- and post-exercise MRI protocol in patients with confirmed CECS compared with normal control subjects (*p* = 0.001).

To our knowledge, this study was the first to make use of SIRs to evaluate for the presence of CECS. We believe that in the absence of more expensive T2W mapping software, this method is an excellent surrogate to objectively measure signal changes in musculature when using a pre- and post-exercise imaging protocol. The statistically significant difference in control and patient groups suggests that this method can be used to accurately diagnose the condition. The findings in this study are in concordance with current literature regarding the normal physiological changes expected in normal muscle during exertion, as well as the pathological changes that occur in patients with CECS.^2,13,15,17,19,28,29,30^

Although the study sample was small, the statistically significant differences in SIR provide a means to objectively evaluate MRI findings in patients where CECS is expected. In some instances, we experienced an overlap in SIR between the patient and control group; however, assessing the interquartile range, no overlap existed between the two groups. Mean SIs of 1.57 and 1.30, respectively, for the patient and control groups were found, and we suggest a correlation with these values when evaluating patients for CECS of the forearm.

The fairly equal SI distribution between the left and right arms likely suggests that no specific predilection exists; however, larger sample groups are required to evaluate this further. The three main muscles involved in our study were the ECR (27.9%), FDP (25.6%) and FDS (23.3%). These muscles were the dominant muscles affected in both the patient and the control groups. Repetitive concentric hand squeezes were undertaken by both the patient and control groups as part of their imaging protocols, which logically explain the involvement of the two large flexor muscle groups; however, the frequent involvement of the ECR muscle was a surprising finding. We believe that this may be because of the continual opposition forces applied by this extensor muscle in maintaining wrist and hand position and stability in the presence of strong flexor forces.

To a smaller degree, the BR (4.7%), AP (4.7%), ECU (2.3%), ED (2.3%), flexor pollicis longus (2.3%) and supinator (4.7%) muscles were occasionally involved. The muscles affected in this study correlate well with the muscles affected in the available literature.^2,13,17,19,29,30^ As all study participants did the same forearm exercise actions as part of their imaging protocol, it is unclear whether this distribution of muscular involvement is specific to the disease process or simply to the exercises performed. Further studies utilising different methods of exerting patients between pre- and post-exercise studies are necessary to evaluate patterns of muscle involvement.

The main limitation of this study is the small sample size. The rarity of the condition as well as our strict inclusion and exclusion criteria limited the number of candidates eligible for the study. Secondly, the control group consisted only of female athletes. This was a complete chance occurrence because of potential male candidates not fulfilling the eligibility criteria. To our knowledge, only a single previous study compared differences in male and female athletes, and the differences reported between the sexes were of dubious significance.^30^ Further research in this area is necessary to determine whether true differences exist between muscle physiology in male and female athletes. As this study evaluated retrospective data collected over 9 years, differences in equipment and imaging protocols can be expected. The control group also had their forearms scanned simultaneously because of the time constraints in the MRI suite used. We believe that as we were comparing pre- and post-exercise studies performed on the same day with the same machines and protocols, this should have no significant outcome on our results. The control group was all investigated on a 3-T MRI machine. To our knowledge, there are no studies comparing the accuracy between 1.5T and 3T in diagnosing muscle oedema. The 3T machines can lead to higher signal-to-noise ratios, shorter scanning times and thinner slices, all of which should not make a difference in detecting muscle oedema.^33^

## Conclusion

This study confirms that SI changes are present in the forearms of both patients confirmed with CECS of the forearm as well as in healthy control subjects who partake in similar high-intensity forearm activities. In patients in whom CECS of the forearm is clinically suspected, we suggest a pre- and post-exercise MRI protocol as part of the patient work up. By calculating the SIR when using a post:pre-exercise imaging protocol, we were able to identify a statistically significant, objectively measured difference between patients and controls. We propose calculation of a SIR to assist in diagnosis of CECS. Further studies with a larger sample size are required to determine specific inclusion and exclusion values.

Future research in the field should include studying a larger number of patients and control subjects to further validate these results. The inclusion of study subjects participating in a greater variety of forearm activities will augment the relevance of our findings.
